# Medical Department of the University of Chicago

**Published:** 1875-07-01

**Authors:** 


MEDICAL DEPARTMENT OF THE UNIVERSITY OF CHICAGO.—We understand that the Rush Medical College, of this city, has effected a permanent union with the Chicago University, and will from this time forth be known as the Medical Department of the latter institution.

